# Case Report: Palmaris Brevis Syndrome Resulting From Acute Wrist Hyperextension

**DOI:** 10.1177/22925503251336249

**Published:** 2025-05-14

**Authors:** Austin E.G. McGrath, Jessica L. Robb

**Affiliations:** 1 Division of Plastic Surgery, Department of Surgery, University of Saskatchewan, Saskatoon, SK, Canada

**Keywords:** acute hyperextension, palmaris brevis syndrome, palmaris brevis, pregnancy, case report, Mots-clés, hyperextension aiguë, syndrome du court palmaire (SCP), muscle court palmaire, grossesse, rapport de cas

## Abstract

A 24-year-old, 19-week pregnant female presented to a community hospital emergency department following an acute hyperextension injury to the left wrist. The pertinent findings on the initial examination included pain, swelling of the ulnar aspect of her palm, and decreased, painful flexion of her small finger. The emergency room physician suspected a flexor tendon injury, and urgent referral to a tertiary level plastic surgery center was made. After plastic surgery evaluation, it was determined that her symptoms were related to the ulnar nerve opposed to an acute tendon injury. After ruling out common surgical pathologies, the diagnosis of palmaris brevis syndrome was provided. Palmaris brevis syndrome does not typically present acutely or post-trauma, and thus is not commonly observed by emergency physicians or acute care hand surgeons. This atypical presentation highlights that palmaris brevis syndrome should be considered in the differential diagnosis of new onset ulnar-sided hand pain.

## Résumé

Une femme de 24 ans, enceinte de 19 semaines, s’est présentée au service des urgences d’un hôpital général à la suite d’une lésion d’hyperextension aiguë de son poignet gauche. Les constatations pertinentes de l’examen initial ont été la douleur, l’enflure de la face ulnaire de sa paume et une flexion douloureuse et limitée de son petit doigt. Le médecin du service des urgences a suspecté une blessure du tendon fléchisseur et a orienté la patiente en urgence vers un centre tertiaire de chirurgie plastique. Après évaluation en chirurgie plastique, il a été établi que ses symptômes étaient liés au nerf ulnaire et non à une lésion tendineuse aiguë. Après élimination des pathologies chirurgicales courantes, le diagnostic de syndrome du muscle court palmaire a été retenu. Le syndrome du court palmaire ne se présente pas habituellement de manière aiguë ou post-traumatique; il est par conséquent rarement observé par les médecins d’urgences ou les chirurgiens de la main en soins d’urgence. Cette présentation atypique souligne que le syndrome du court palmaire doit être envisagé comme diagnostic différentiel en cas de survenue récente d’une douleur du côté ulmaire de la main.

## Introduction

The palmaris brevis (PB) muscle is located superficial to the hypothenar eminence.^1^ It originates from the palmar aponeurosis and inserts into the dermis of the hypothenar region of the hand.^1^ The PB aids the ability to grip through tightening the palmar aponeurosis, deepening the cup of the hand.^1^ The innervation is from a superficial terminal branch of the ulnar nerve, originating distal to Guyon's canal and traveling deep to the PB muscle.^2^ This superficial terminal branch terminates as 2 palmar digital nerves supplying the ulnar aspect of D4, and both the medial and ulnar aspect of D5.^2^ Importantly, the branch supplying the PB is the only motor component of the superficial branches of the ulnar nerve.

Throughout pregnancy the female body undergoes several normal physiological changes affecting nearly every system of the body; of most relevance for this discussion is the alteration of fluid status.^3^ There is increased aldosterone and cortisol production which increases fluid retention. This increase results in more fluid entering interstitial spaces and compartments, and elevated pressure in the tissues can result in nerve compression syndromes, most commonly Carpal tunnel syndrome (CTS).^3^

Palmaris brevis syndrome (PBS) is a pseudodystonia resulting in involuntary contractions of the PB muscle, believed to be a result of compression irritation of the motor nerve branches from the ulnar nerve. The other cases of PBS in the literature result from repetitive, manual use of the hands or frequent use of vibratory tools.^4,5^ Most patients with PBS are referred nonurgently to movement disorder clinics.

This case report documents a 24-year-old female that experienced an acute hyperextension injury to the left wrist resulting in abrupt onset of decreased and painful flexion of D5, paresthesia in the ulnar innervated digits, and muscle spasm in her ulnar hand. She was diagnosed with PBS through clinical history and exam in combination with EMG studies after other possibilities were ruled out. This case highlights the possibility of acute onset following trauma, therefore emergency physicians and hand surgeons should be aware and consider it within the differential for similar presentations.

## Case Report

At the time of presentation, the patient was a 24-year-old, left hand dominant, 19-week pregnant female who was referred to University Plastic Surgery service for suspected flexor tendon injury following an acute hyperextension of the left wrist when her son attempted to jump on her in bed and she defended with her hand. She was an otherwise healthy individual with no history of a muscular diseases or nerve compressions/ syndromes. There was immediate pain and edema to the ulnar hand following the injury, prompting the individual to seek immediate care at her local emergency department. Examination by an emergency physician documented limited active flexion of D5 and associated pain and swelling of the hypothenar region of her left hand. With this presentation, specifically the limited flexion, the emergency physician suspected a flexor tendon injury and referred her to a tertiary care center for surgical assessment.

Upon formal hand surgery assessment, her exam was notable for abduction of the D5 at rest, decreased active range of motion of D4 and D5, pain with resistance of flexion in D4 and D5, subjectively decreased sensation in the ulnar nerve distribution, and a positive Tinel's sign over Guyon's canal. Her tenodesis was intact, and her exam was not in keeping with a flexor tendon injury. There was a firmly contracted and easily palpable muscle on the hypothenar side of the hand. The working diagnosis included a hook of hamate fracture, dystonia of the hand or nerve compression syndrome, but further evaluation was required to rule out an acute pathology requiring surgical intervention.

Initial hand and wrist radiographs showed no evidence of a hook of hamate fracture. Ultrasound found no evidence of tendon or nerve damage, and no identifiable source of nerve impingement. With no obvious tendon or bony injury revealed, the patient was placed in a volar wrist and soft elbow splint and was asked to return in 1 week's time for reassessment.

Based on the appearance of her hand and a literature review the working diagnosis PBS was made. EMG demonstrated cramp-like discharges in her PB; this finding, along with her clinical presentation, confirmed the diagnosis of PBS.

The most effective treatment noted in the scarce literature available is neurotoxin injections of PB for symptomatic relief. Due to the patient's pregnancy, she did not want to undergo treatment with neurotoxins and further management was deferred until the postpartum period.

**Figure 1.**
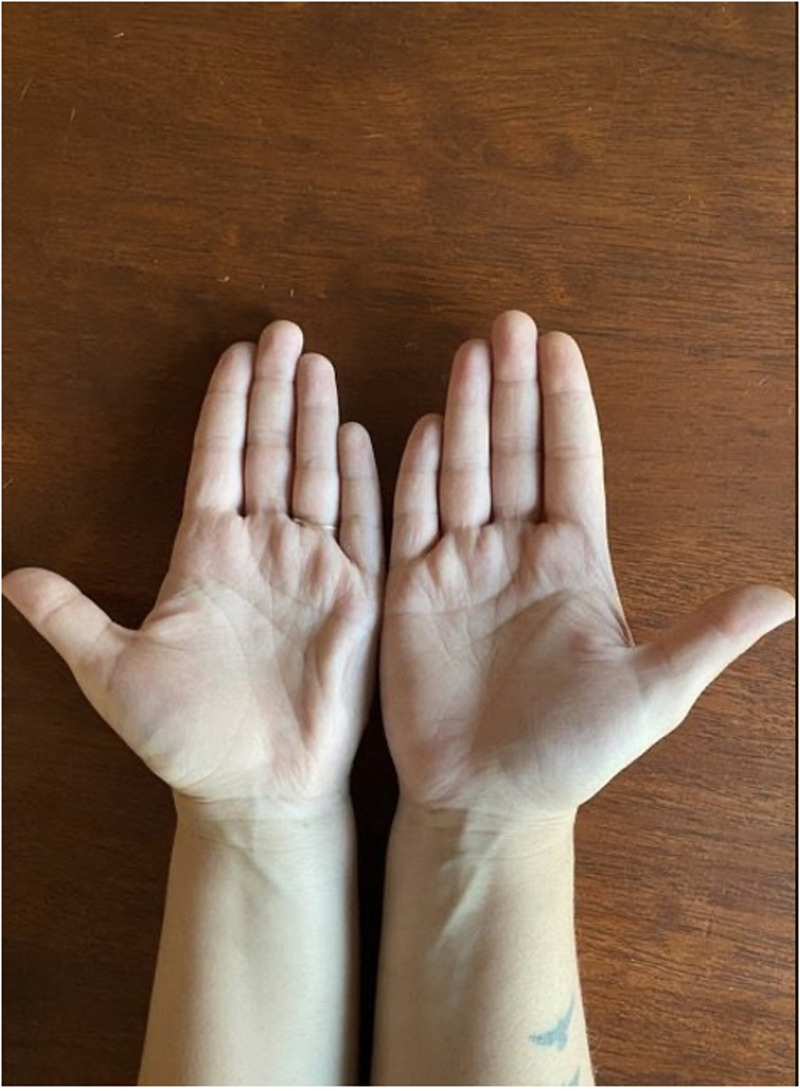
Anterior view of the patient’s hands demonstrating continued furrowing of left hypothenar region, consistent with palmaris brevis syndrome (PBS).

**Figure 2.**
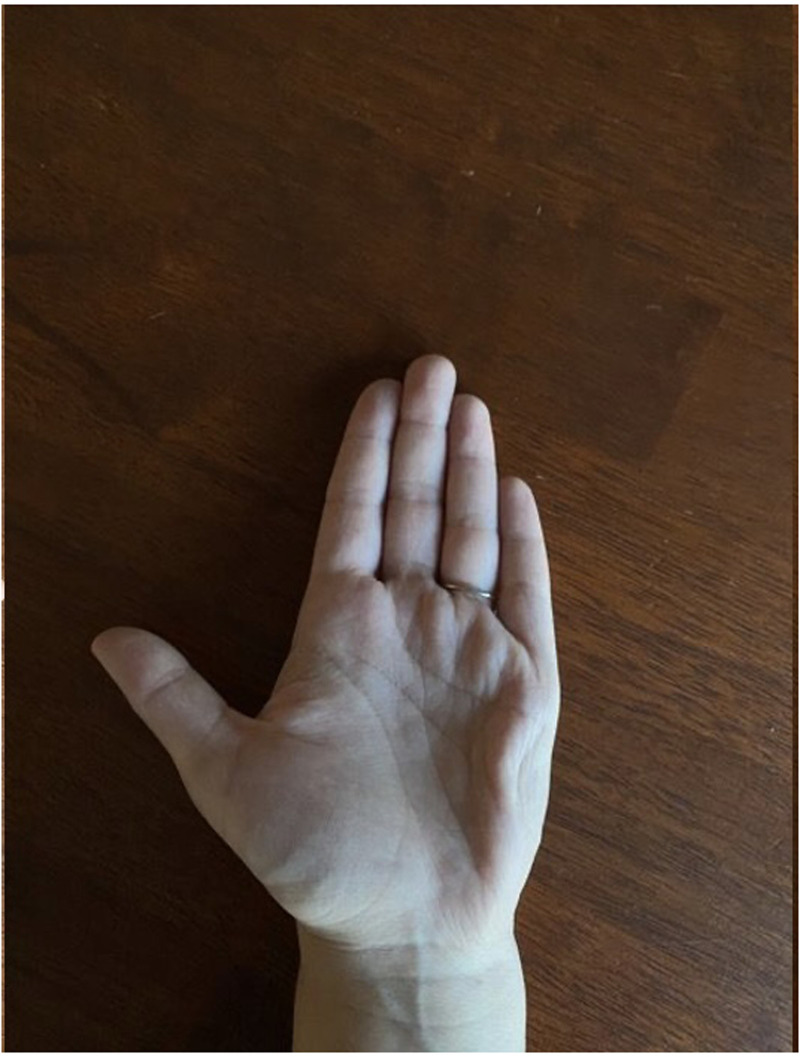
Close up image of left hand alone demonstrating dystonia of hypothenar region.

**Figure 3.**
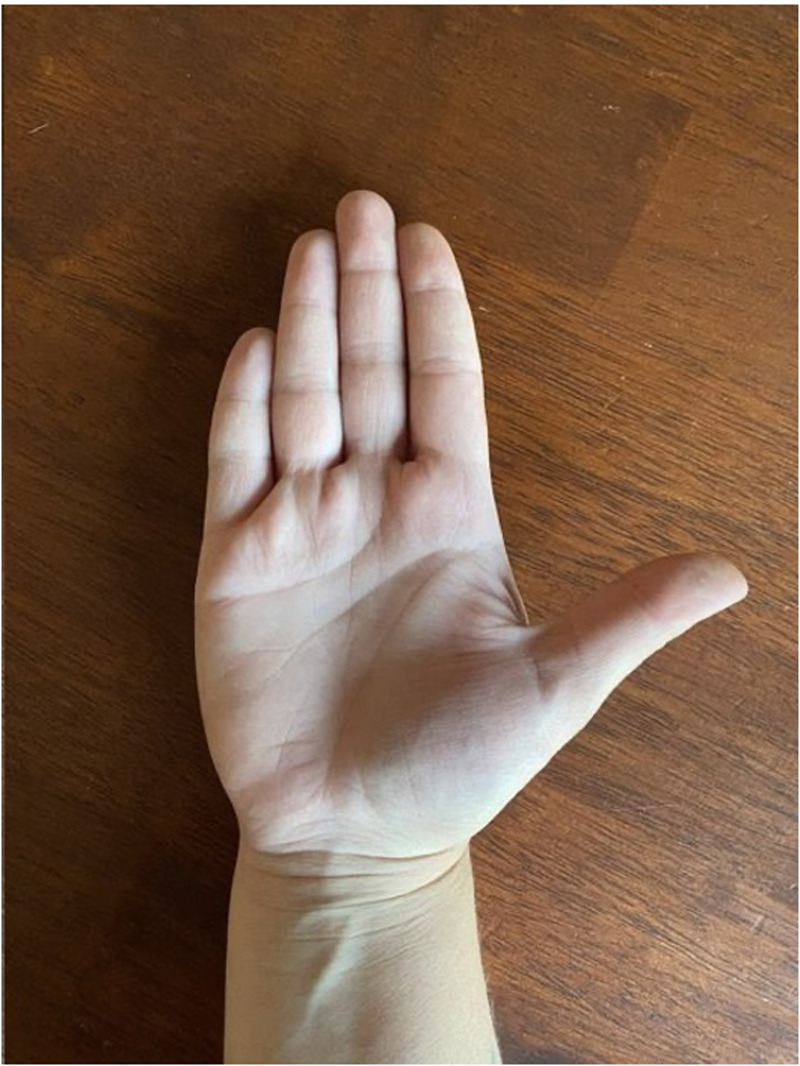
Close up image of normal right hand.

Eighteen months after the initial injury (14 months postpartum) the only remaining symptom was the dystonia of the PB of her left hand (Figures 1–3), with the other symptoms resolving within the initial 1 to 2 months postinjury. She was performing her activities of daily living with minimal functional limitations; however the contraction of her hypothenar muscles increased with activity. At this point, she is continuing conservative management while awaiting coordination of neurotoxin injections.

## Discussion

PBS is a rare condition, with minimal cases reported in the literature. Furthermore, this case had an atypical presentation due the acute mechanism of injury, her pregnancy, and the constant dystonia. While there is already a lack of knowledge surrounding PBS, these additional atypical aspects make this case particularly unique.

A complicating factor surrounding this case was her pregnancy. Particularly relevant physiological changes of pregnancy within this case are the impacts on the nervous system, including compression within compartments and resultant paresthesias and reduced function.^6^ A primary question was if her pregnancy was influencing her susceptibility to PBS following her injury. However, the persistence of her symptoms after her delivery makes it less likely that her pregnancy was directly related to her presentation. Her pregnancy did impact her ability/willingness to treat her PBS with neurotoxins due to potential risks for the fetus.

## Conclusion

This case documents a unique case of post-traumatic acute onset of PBS in a pregnant female. PBS characteristically presents as painless involuntary contractions secondary to overuse that do not result in disability.^7^ In this instance, the patient had a preceding trauma and presented to a surgical service with pain, paresthesia, decreased range of motion, in addition to a contraction of her hypothenar muscles. This case will add information to better understand this rare condition and document the possibility of post-traumatic presentation.
